# Betel Chewing and Nonalcoholic Steatohepatitis

**DOI:** 10.7759/cureus.2943

**Published:** 2018-07-08

**Authors:** Wissam Bleibel, Saad Saleem

**Affiliations:** 1 Gastroenterology, Mercy St. Vincent Medical Center, Toledo, USA; 2 Internal Medicine, Mercy St. Vincent Medical Center, Toledo, USA

**Keywords:** betel, metabolic syndrome, nonalcoholic steatohepatitis, diabetes mellitus

## Abstract

Betel chewing is a common social practice in many regions of the world particularly in Southeast Asia and among the Asian immigrant populations in the West. Several studies have shown betel chewing to be associated with increased risk for various health complications including liver cirrhosis and hepatocellular carcinoma. The exact mechanism by which betel causes liver damage has not been elucidated. We present a 31-year-old Asian immigrant in the United States of America (USA) with no family history of the liver disease and non-smoker who was found to have an unexplained persistent mild elevation of liver transaminases. She reported more than 16 kilograms of weight gain over an eight-year period in association with heavy betel chewing. Despite diet and exercise, she was not able to lose weight. Besides, she developed dyslipidemia and gradual worsening of glucose tolerance. Liver biopsy showed severe steatosis with features of nonalcoholic steatohepatitis (NASH). The gradual development and worsening of metabolic syndrome and NASH paralleling betel use are very indicative of the hepatic steatosis being caused by betel.

## Introduction

Betel chewing is a common social practice in many regions of the world particularly in Southeast Asia. Over 600 million people chew betel products. This is an ancient practice that originated in the Indian Subcontinent and slowly moved to the Western society with the migration of millions of people [1]. Betel (also known as Paan or Gutka) is the fourth most commonly used psychoactive substance after caffeine, nicotine, and alcohol. In addition to its psychoactive effect, betel is commonly used in folk medicine. Betel use involves oral placement of shards of Areca nut wrapped with slaked lime in a betel leaf. Additives to this mixture include spices, sweeteners, or tobacco. The active ingredients dissolve and get absorbed slowly through the buccal mucosa [2, 3].

Studies from the United States of America (USA) and the United Kingdom (UK) where the South Asian Community is large and rapidly growing have shown that betel products are readily available and commonly used in ethnic enclaves. In the USA, this product is legal and inexpensive thus 20% to 60% of Asian Americans use betel while its use is very uncommon among the other racial groups [2-4]. Several studies have shown betel chewing to be associated with increased risk for various medical conditions including oral cancer, type II diabetes mellitus (DM), hypertension, hyperlipidemia, coronary artery disease, liver cirrhosis (LC), and hepatocellular carcinoma (HCC). The exact mechanism by which betel causes liver damage has not been elucidated [5-9]. We report a case of severe nonalcoholic steatohepatitis (NASH) that developed in an Asian American patient who habitually chewed betel leaf with betel nuts.

## Case presentation

A 31-year-old Asian American female with past medical history of chewing of betel leaf with betel nuts and non-smoker, who immigrated to the USA from Burma four years previously, was referred to the Digestive Health Center at the University of Virginia for evaluation of elevated transaminases discovered upon routine testing. She reported no previous history of liver test abnormality or liver disease. She was not using any hepatotoxic prescription or over the counter medications or supplements and reported rare consumption of alcohol. Besides, she had no family history of liver disease, hepatocellular carcinoma, autoimmune disorders or diabetes mellitus.

The physical examination was significant for obesity with weight 66 kg, height 146 cm, body mass index (BMI) 31, hepatomegaly, nonpalpable spleen, and lack of stigmata of chronic liver disease. Extensive laboratory workup revealed normal complete blood count, renal function, serum electrolytes, iron studies, serum immunoglobulin levels, and ceruloplasmin. Also, hepatitis B and C serologic tests and autoimmune markers were negative. Fasting lipid profile revealed dyslipidemia (total cholesterol 260 mg/dL, triglycerides 267 mg/dL, high density lipoprotein 45 mg/dL, and low density lipoprotein 170 mg/dL). Fasting blood glucose was 165 mg/dL with simultaneous fasting insulin level of 4.8 mill international units/liter and a homeostasis model assessment of insulin resistance score of 2.9, thus has insulin resistance. Hemoglobin (Hgb) A1c level at this time was 6.8% which was higher than previous values from eight months prior (6.1%), 16 months prior (6.1%) and 39 months prior (4.8%).

**Figure 1:**
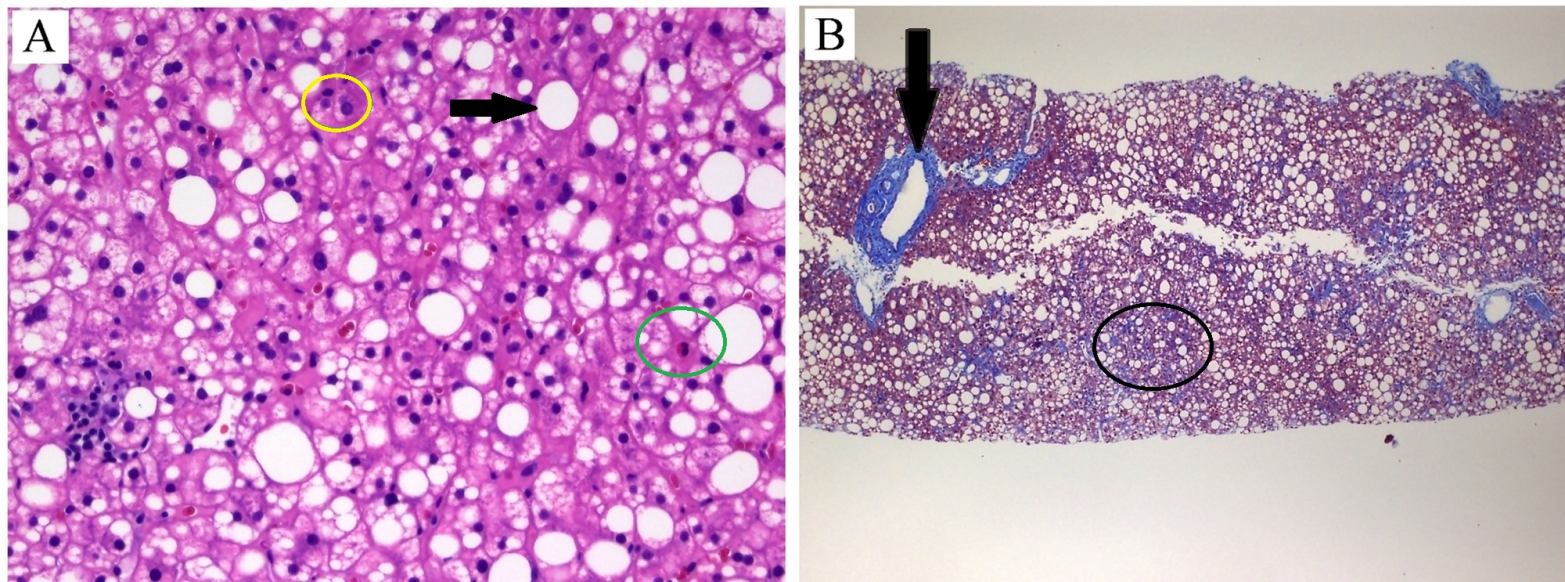
Histopathologic findings. Panel A is a high power (40x) view of hematoxylin and eosin stain that shows hepatic steatosis (black arrow) with lymphocytic (yellow circle) and rare neutrophilic (green circle) inflammation. Panel B is a low power (4x) view of trichrome stain showing periportal (black arrow) and perisinusoidal fibrosis (black circle). Courtesy of William A. Kanner M.D., Department of Pathology, University of Virginia, Charlottesville, VA.

Ultrasonographic evaluation of the abdomen revealed hepatomegaly, hyperechogenic liver indicative of severe hepatic steatosis, an ill-defined liver mass, and standard spleen size. Magnetic resonance examination defined the liver mass as hemangioma in addition to hepatomegaly and severe hepatic steatosis. Ultrasound-guided liver biopsy (sample size of 3.5 cm) showed severe macrosteatosis with mild lobular and periportal inflammation associated with focal hepatocyte damage (Figure 1, Panel A). Trichrome staining revealed focal portal, periportal and perisinusoidal fibrosis consistent with stage II (Figure 1, Panel B) with a NASH activity score (NAS) of 7. The patient was instructed to exercise routinely and follow a healthy diet. She was seen in follow-up for three months at which time her weight was unchanged. Physical examination revealed dark brownish red pigment on the teeth, tongue, and oral mucosa. Upon questioning the patient about this finding, she admitted to chewing betel on an average of 10 times per day for the last eight years. The patient reported an associated weight gain of around 16 kg from her baseline weight maintained for many years at 50 kg (BMI 23.5). This significant weight gain could not be reversed despite daily physically demanding work and following a healthy diet recommended by her primary care physician.

## Discussion

Betel chewing is an ancient social habit with 10 to 25% of the world population using this psychoactive agent. Although the highest concentration of betel users is in Southeast Asia, reports from western countries have shown that this habit is practiced by a significant proportion of specific Asian Immigrant populations in the USA and the UK [2, 3]. Several studies have shown that the chronic use of betel leads to multiple health issues. In addition to many extrahepatic complications, betel chewing has been associated with elevated liver function tests, and increased risk of LC and HCC yet the mechanism has not been clarified [6-13]. Wu et al. demonstrated a 4.25-fold increase in the risk of LC and HCC in current betel chewers and a 1.89-fold increase in ex-chewers in comparison to never-chewers. The study revealed a dose-dependent effect for quantity, duration and cumulative exposure of chewing. Also, betel chewing had an additive, synergistic effect on hepatitis B and C-related risks for LC and HCC [13].

Several studies have shown a significant increase in the prevalence of metabolic syndrome (MS) and type II diabetes mellitus (DM) among betel chewers in Asia [5, 14] and Asian immigrant communities in the West [15, 16]. In a study from Taiwan, Yen et al. showed that the odds ratios for MS were 1.38 and 1.78 in ex-chewers and current chewers, respectively, even after adjustment for other significant correlates such as family history of hypertension and DM. Also, Chung et al. demonstrated that in comparison with non-chewers, areca nut chewers have higher age-adjusted prevalence rates of hyperglycemia (11.4% vs. 8.7%) and type II DM (10.3% vs. 7.8%). The hyperglycemic and diabetogenic effects of betel chewing were both dose and duration dependent [17].

MS is strongly associated with nonalcoholic fatty liver disease (NAFLD) and its subtype NASH. The prevalence of NAFLD has dramatically increased, and it is currently considered to be the most common nonviral liver disease affecting up to 40% of the Western and around 30% of the Asian-Pacific populations [18]. Although the exact mechanism of liver damage caused by NASH has not been fully elucidated, this condition is known to cause cirrhosis and hepatocellular carcinoma. The most widely accepted theory implicates insulin resistance as the principal mechanism leading to chronic inflammation in the liver with resultant fibrosis and eventual cirrhosis [19].

The exact mechanism via which betel leads to the development of metabolic syndrome and other health complication is also not entirely clear. Nitrosated derivatives of betel alkaloids have been shown to enhance appetite and to cause increases in circulating markers of inflammatory and cardiovascular damage including tumor necrosis factor-α (TNF-α), interleukin-6, reactive oxygen species, and expression of active nucleate factor-κB [20].

Our patient developed gradual and progressive manifestations of metabolic syndrome in association with an elevation in transaminases and ultrasonographic suggestion of fatty infiltration. Over a period of eight years, she progressively gained significant weight, developed hypertension, dyslipidemia, impaired glucose tolerance and eventually diabetes mellitus in association with her heavy use of betel. The lack of family history of metabolic syndrome and other conditions or medications that could be blamed for her metabolic derangement suggests that betel is the causative factor of her MS and the underlying NASH. Interestingly, most of her weight gain occurred in her first four years of betel use yet despite the much milder weight gain over the last 3.5 years (a total of 3.5 kg) her glucose tolerance dramatically worsened as indicated by the gradual increase in Hgb A1C from 4.8 to 6.8%. Besides, despite the high fasting glucose level and Hgb A1C, her fasting insulin level was normal with resultant Homeostasis Assessment Model (HOMA) score of 1.96. This could be indicative of some degree of an insulin-sensitizing effect of betel.

Our case suggests that the liver disease associated with betel chewing might be caused by NASH. This is supported by the many studies from Asia reporting an increased prevalence of metabolic syndrome, as well as liver function test abnormality, cirrhosis, and hepatocellular carcinoma in betel chewers.

## Conclusions

Betel chewing is a common practice in many countries in Asia and among immigrant populations in the West. Studies have shown a strong association between betel and development of cirrhosis and hepatocellular carcinoma yet the exact mechanism via which betel leads to liver damage is not clear. We suggest NASH as the underlying cause of liver disease in betel chewers. Further studies are required to confirm this theory. We also suggest that patients with NASH should be asked about betel use as an underlying cause of their metabolic and hepatic derangement.
